# Interaction of POPC, DPPC, and POPE with the μ opioid receptor: A coarse-grained molecular dynamics study

**DOI:** 10.1371/journal.pone.0213646

**Published:** 2019-03-14

**Authors:** Marie-Ange Angladon, Mathieu Fossépré, Laurence Leherte, Daniel P. Vercauteren

**Affiliations:** Laboratoire de Physico-Chimie Informatique, Unité de Chimie Physique Théorique et Structurale, Namur Medecine and Drug Innovation Center (NAMEDIC), Namur Research Institute for Life Sciences (NARILIS), University of Namur (UNamur), Namur, Belgium; University of Cambridge, UNITED KINGDOM

## Abstract

The μ opioid receptor (μOR), which is part of the G protein-coupled receptors family, is a membrane protein that is modulated by its lipid environment. In the present work, we model μOR in three different membrane systems: POPC (1-palmitoyl-2-oleoyl-sn-glycero-3-phosphocholine), POPE (1-palmitoyl-2-oleoyl-sn-glycero-3-phosphoethanolamine), and DPPC (1, 2-dipalmitoyl-sn-glycero-3-phosphocholine) through 45 μs molecular dynamics (MD) simulations at the coarse-grained level. Our theoretical studies provide new insights to the lipid-induced modulation of the receptor. Particularly, to characterize how μOR interacts with each lipid, we analyze the tilt of the protein, the number of contacts occurring between the lipids and each amino acid of the receptor, and the μOR-lipid interface described as a network graph. We also analyze the variations in the number and the nature of the protein contacts that are induced by the lipid structure. We show that POPC interacts preferentially with helix 1 (H1) and helices H5-H6, POPE, with H5-H6 and H6-H7, and DPPC, with H4 and H6. We demonstrate how each of the three lipids shape the structure of the μOR.

## Introduction

Opioid receptors, whose crystallographic structures were revealed in 2012 [1–3], are part of the largest group of integral membrane proteins, the G protein-coupled receptors (GPCRs) superfamily [4]. The structure of GPCRs consists of seven α-helices (H1-H7), followed by a short helix (H8), separated by three extracellular loops (EL1-EL3), and three intracellular loops (IL1-IL3). The N-terminal and C-terminal domains are located at the extra- and the intracellular side, respectively. The opioid receptors are divided in three subtypes: μ, δ, and κ. Protein μ is the most important for the treatment of pain with morphine and opiate alkaloids. These drugs are addictive and their clinical efficacy is limited by side effects such as dependence and tolerance [5].

Protein/lipid interactions influence the membrane protein functions through conformational changes [6–9]. There are thousands of lipids in a plasma membrane, and properties such as their length, the saturation, and the nature of the head groups are essential to understand their functions [10]. For example, several biochemical experiments demonstrated the role of cholesterol on the GPCRs [11, 12]. Particularly, Zheng and its collaborators [13] considered the interactions between cholesterol and the μ opioid receptor (μOR) signaling complex with a new method involving an antibody. The crystal structure of μOR crystallized with cholesterol has been solved in 2012 [2]. To crystallize a membrane protein, it is necessary to remove most of the surrounding lipids. Only the tightest bound lipids are found in the final structure, as illustrated by Kobilka and Schertler for the β2 adrenoceptor and rhodopsin [14]. The location of weaker lipid binding sites is therefore usually observed using classical molecular dynamics (MD).

To apprehend interactions between membrane proteins and lipids, MD has indeed been shown as a theoretical method of choice [15, 16]. As examples, one can cite the MD studies focused on the well-known rhodopsin receptor embedded in a membrane composed of phosphatidylcholine, phosphatidylethanolamine, and cholesterol [17, 18]. Both researches demonstrated the role of the lipids on the structure and function of the rhodopsin receptor. Since several years, MD methods have thus largely proven their efficiency in the investigation of GPCRs [19–22] and they are proven their efficiency as they reproduced experimental results [23].

To reach longer simulation times, the MARTINI coarse-grained (CG) model is often preferred to all-atom. Many simulations are made with coarse-grained model to study GPCRs-lipid interactions, as cholesterol binding sites for serotonin 1A [24]. Rhodopsin dimerization interfaces have been observed and confirmed by biochemical cross-linked experiments [25]. Recently, Marino *et al*., performed CG MD simulations with active and/or inactive μOR in multi-component membranes [26]. The type of μOR interfaces are induced by the shape complementarity between conformations, indirect lipid effects play a role in receptor oligomerization.

In the present paper, we apply CG classical MD simulations to study μOR-lipid interactions over long simulation times. The speed of calculation time in CG allows to conduct long simulations and repeat them which is necessary for the analyze of protein-lipid interactions. We consider μOR embedded in three types of membranes: 1-palmitoyl-2-oleoyl-sn-glycero-3-phosphocholine (POPC), 1-palmitoyl-2-oleoyl-sn-glycero-3-phosphoethanolamine (POPE), and 1, 2-dipalmitoyl-sn-glycero-3-phosphocholine (DPPC). These three lipids are choosen in accordance with the majority proportion of lipids in the plasma membrane. Phospholipids represent near of 50% of the lipids and among them, phosphatidylcholine and phosphatidylethanolamine are the main ones [10]. μOR-POPC will serve as the reference system for comparisons with DPPC, a saturated lipid, and POPE, which presents a different head group, *i*.*e*., ethanolamine. The POPE membrane highlights the role of the head group on the positioning of the acyl chain against the protein, while the saturated lipid, DPPC, constrains the receptor and hence promotes contacts between the lipids and the protein. Our results, put together, demonstrate the crucial role of the membrane composition on the conformational changes of μOR.

## Materials and methods

### Molecular systems

**Fig 1:**
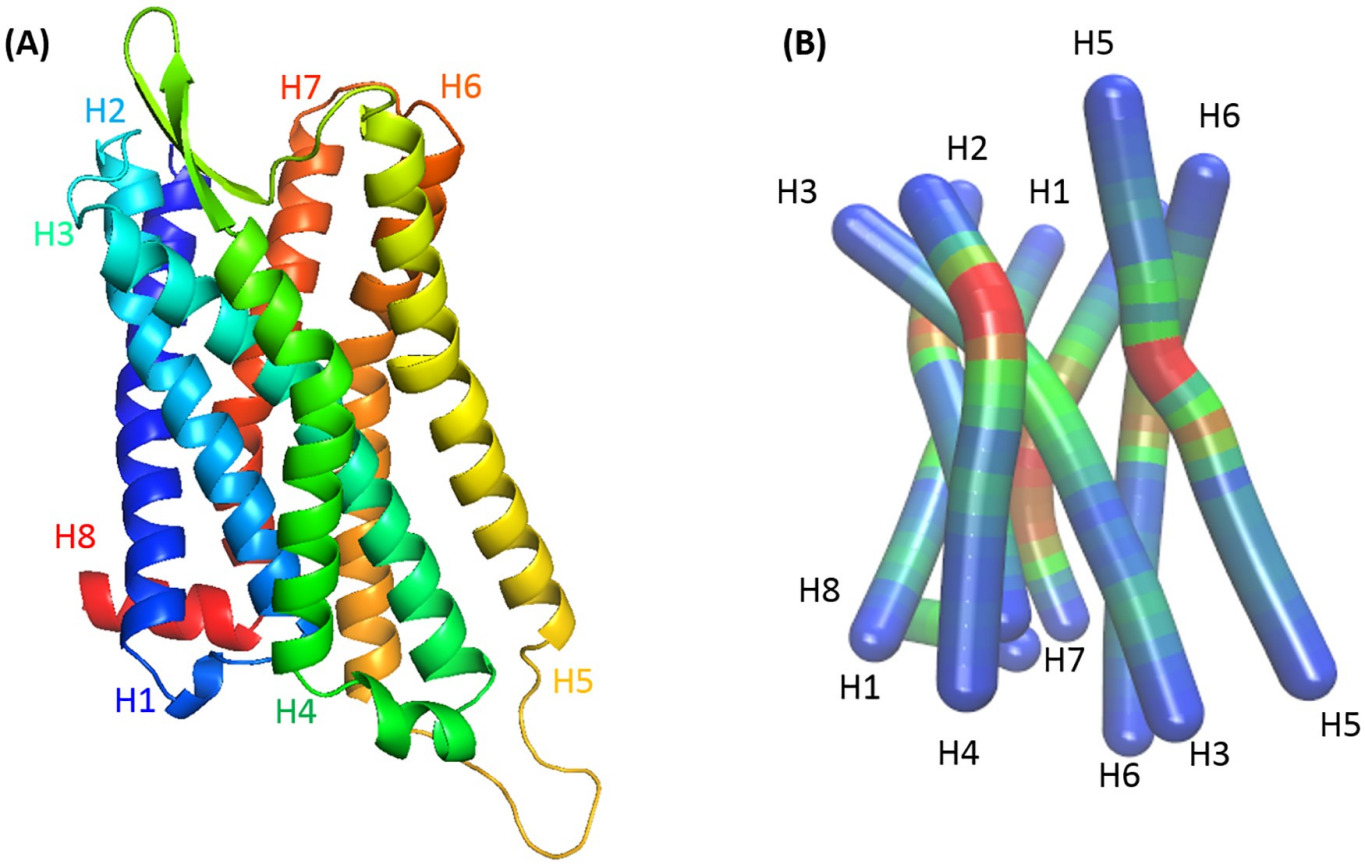
Schematic structure of μOR. Notations of the 7 transmembrane helices (H1 to H7) and the intracellular helix H8. **(A)** Ribbon representation. **(B)** Bendix representation, ranging from blue (no helix tilt) to red (maximum helix tilt).

The complete all-atom structure of μOR, already described in [27], was obtained from the X-ray diffraction structure [2] available in the PDB (ID: 4DKL) and further modelled with the CHARMM22 in conjunction with CHARMM36 force field (FF) [28]. The protein is composed of 7 transmembrane helices (H1 to H8) and an intracellular helix 8 (Fig 1). H3 is located between H2 and H4 in the extracellular and between H4 and H5 in the intracellular part. Helix H5 is divided into two parts by the P244 which induces a torsion angle.

To set up the supramolecular protein-lipid system, three membrane models, constituted of POPC, DPPC, or POPE molecules were generated with the Charmm Builder [29]. The μOR receptor was then embedded in each membrane composed of 600 lipids, solvated with water (25 Å above and below the membrane) and neutralizing counterions (13 Cl^-^), for a simulating box of 200 x 200 x 90 Å with VMD scripts [30] A comparison in terms of AA and CG systems of all three considered systems is given in S1 Table.

**Fig 2:**
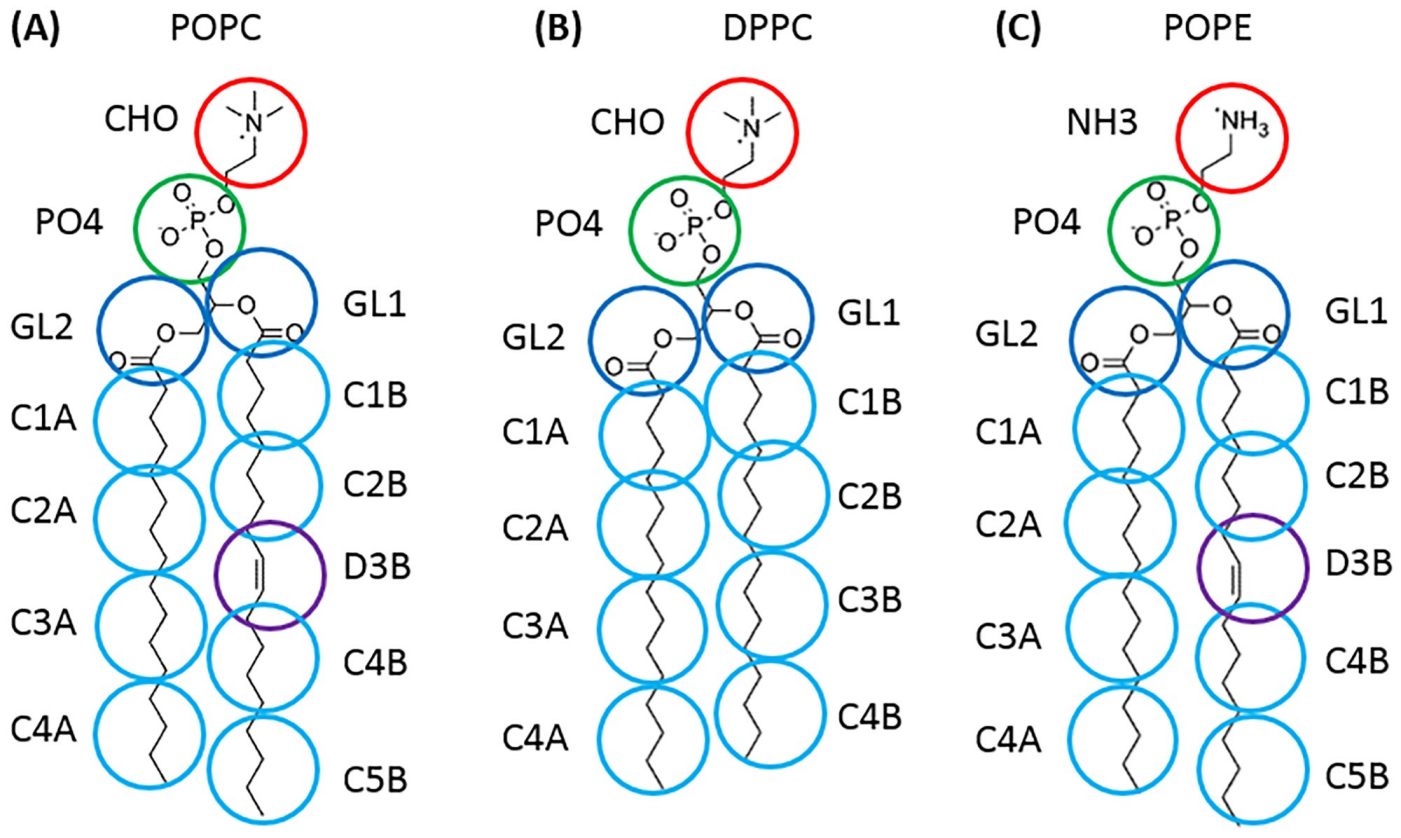
Superimposition of the heavy atoms and MARTINI beads of lipid. **(A)** POPC, **(B)** DPPC, and **(C)** POPE.

The so-obtained AA systems were then transformed into MARTINI v.2.1 CG representations, using VMD [31–33]. In the MARTINI FF, there are four basic interaction sites: polar (P), nonpolar (N), apolar (C), and charged (Q) [33]. POPC is modelled here with a 13-beads model with two Q beads representing the zwitterionic head groups, two N beads for the ester groups, and four to five C beads for the two fatty acyl chains (Fig 2A). DPPC differs from POPC by one C bead in one of the two acyl chains (Fig 2B). The head group of POPE differs from POPC as it includes an ethanolamine bead (Fig 2C). DPPC and POPC have similar heads but different tails; in POPC, the D3B beads correspond to an unsaturation in tail B (Fig 2A), whereas in DPPC, both saturated tails involve a C3 bead (Fig 2B). DPPC is a smaller lipid than POPC and POPE with no C5B bead.

### Molecular dynamics simulations

The CG MD simulations were performed with the NAMD 2.8 package [34]. To remove initial unfavorable contacts, the total potential energy of the systems was first minimized using a conjugate gradient approach with a maximal number of 1,000 steps (Table 1). Then, a series of equilibration steps using Langevin dynamics was performed to further remove contacts that led to high energy values, first during 20 ns with a time step of 10 fs in NVT conditions, and then during 3 x 10 ns with a progressively increased time step value, *i*.*e*., 10, 20, and 30 fs, in NPT conditions. The equilibration was needed to melt the lipid tails and let the lipids rearrange correctly in the membrane and around μOR. Finally, the μOR-POPC, μOR-DPPC, and μOR-POPE systems were simulated for 5 μs MD at P = 1.01325 bar and T = 310 K. Each production run was repeated three times and results were averaged for statistical purpose.

**Table 1:** Protocol of the coarse-grained MD simulations.

	Integration step (fs)	Time	Ensemble
**Minimization**	1,000 steps		
**Equilibration**	10	20 ns	NVT
	10	10 ns	NPT
	20	10 ns	
	30	10 ns	
**Production**	30	5 μs	

For information, the MD simulation protocol for the AA system is explained in S2 Table.

### Analysis methods

The MD results were analyzed after alignment of all saved system frames on the μOR structure with the program Carma [35]. Due to the rigid MARTINI model, we consider that μOR conformations are sufficiently stable to allow path calculations from MD frames saved every 10^6^ iterations.

The Bendix plugin [36], part of VMD, was used to evaluate the maximum tilt angle of the receptor helices. This plugin uses a sliding window of four residues, along the predefined helix, to give local helix axes that are joined by a spline. It is therefore possible to study helix torsions more realistically. The helices globally rotate around the AAs located in their middle.

In-house Python3 scripts were written to measure the distance between beads of the protein and beads of the lipids. Contacts were indeed established between lipids and protein when the distance is less than 0.6 nm [24]. To consider a contact between two beads in our work, a distance criteria of 0.5 nm was selected as in the work of de Jong *et al*. who also studied protein-lipid interactions using the MARTINI model [37]. In our work, we took into account three distances to study the contacts between the protein and the lipid beads: 0.5, 0.6, and 0.7 nm (S1 Fig). We did not observe any differences between the relative number of contacts at each of the three distance values. As an example, the number of contacts between the POPC beads and F204 amounts to F204 (2,867 contacts below 0.5 nm, 2,329,544 contacts below 0.6 nm, and 3,592,610 contacts below 0.7 nm. Even if the numbers obviously differ, their relative occurrence frequencies yield similar figures. Moreover, on a purely visual aspect, the large number of contacts formed by the most interacting AAs tends to hide the contacts formed by the least interacting AAs. The color contrast is better with a distance contact of 0.5 nm and such a cutoff distance does not lead to any loss of information.

We determined the contact frequency between each amino acid (AA) and each surrounding lipid, each AA being colored by its contact frequency with lipids on the primary structure representation.

Finally, we used Gephi [38] to analyze the lipid sites at the μOR surface. Gephi is a graph visualization software that allows to display results in terms of graphs or networks. We used the default parameters of Gephi and defined the input parameters as follows. For each lipid that interacts with μOR at least 20% of the MD simulation time, helices located at a distance shorter than 0.5 nm were searched, for each MD frame. Each contact was then defined by each helix number. Identical successive helix numbers were deleted to visualize the lipid movements from one visited site to another. The data associated to all lipids were considered to generate networks, as a function of the membrane composition, in order to determine the protein sites visited by the lipids and the paths they follow, represented by the nodes and connections. Both node and path size values are proportional to their browse frequency. Lipid movements in the internal and in the external leaflets of the membrane were separated. In addition, Gephi allows to depict each output network in terms of clusters, as described in the Louvain methods with a modularity coefficient of 0.8 [39].

## Results

### Comparison between AA and CG methods

In this part, we compare the results obtained from a CG and an AA simulation of μOR embedded in a POPC membrane. As the CG model was directly obtained from the AA model, we compare the behavior of the same system with two different simulation methods.

We first conducted AA and CG simulations of the lipid membranes to verify the bilayer properties, before conducting the GPCR-lipid MD simulations. As an example, we observed that the area per POPC molecule is equal to 67.68 Å^2^ for the CG MD simulation, to be compared to the reference value of 68.3 Å^2^ [cfr Web site of membrane builder].

We also calculated the RMSD for μOR and for each helix (Table 2).

**Table 2:** RMSD comparison for each μOR helix embedded in POPC membrane.

Helix	AA begin—AA end	RMSD (AA)	RMSD (CG)
**H1**	65–95	2.37	2.88
**H2**	103–130	1.55	2.86
**H3**	138–169	1.88	2.80
**H4**	180–204	1.65	2.87
**H5**	226–257	1.58	3.36
**H6**	273–304	2.25	4.21
**H7**	312–340	2.28	2.85
**H8**	341–352	1.80	3.46

The RMSD is obtained from the 1μs AA MD run at P = 1.01325 bar and T = 310 K and the 5 μs CG MD runs at P = 1.01325 bar and T = 310 K.

As can be seen in Table 2, the differences between the RMSD from the AA and CG simulations are not significant for the H1, H2, H3, H4 and H7 helices. The deviation is higher for the H5, H6 and H8 helices. Indeed, the initial structure is very constrained because of the crystallization technique as compared to the structures resulting from the MD. In addition, H5 is bent due to the P244, as shown in Fig 1.

Finally, a comparison was made to validate the structural variations by the calculation of the angles of each helix of μOR obtained by both AA and CG simulations. We have chosen to show the starting point with the μOR tilt angles in the crystal structure (Table 3).

**Table 3:** Tilt angle, using Bendix, for μOR embedded in POPC membrane.

Helix	Crystal structure tilt (°)	Average tilt, AA (°)	Average tilt, CG (°)
**H1**	14.95	12.29 ± 2.71	17.1 ± 1.7
**H2**	7.54	14.25 ± 3.73	14.2 ± 1.0
**H3**	9.84	12.09 ± 2.53	15.2 ± 0.8
**H4**	24.03	21.89 ± 5.25	19.5 ± 0.8
**H5**	17.59	22.02 ± 4.56	55.7 ± 7.1
**H6**	17.90	19.50 ± 3.32	17.1 ± 1.6
**H7**	18.92	19.20 ± 2.65	15.2 ± 0.7
**H8**	7.09	7.19 ± 3.25	9.8 ± 0.3

The average tilt angles are obtained from the 1μs AA MD run at P = 1.01325 bar and T = 310 K and the 5 μs CG MD runs at P = 1.01325 bar and T = 310 K.

As mentioned earlier, we focused more on the location of helix H3 than on its tilt value. H3 is located between H2 and H4 in the extracellular part and between H4 and H5 in the intracellular part (Fig 1A). It is particularly important for the understanding of the lipid sites at the protein surface as discuss later. The figures reported in Table 3 show that the mean tilt values calculated from both the AA and CG simulations are comparable to the experimental values. The average tilt of helix H5 is an exception with a value of 55.7° for the CG simulation and 22.02° for the AA one.

In the GPCR family, P244 of H5 is known to induce a strong deformation of the helix. It is therefore necessary to calculate the tilt angle of the part before and after the deformation. Helix H5 consists of two parts, separated by 4 amino acids, A240 to P244, shown in Fig 1B. To better understand the difference in tilt angles between the AA and CG methods, we measured the tilt for the W226—A240 part and the P244—L257 part (Table 4).

**Table 4:** Tilt angle of H5, with standard deviations for the μOR embedded in POPC membrane.

AA begin—AA end (H5)	AA method (°)	CG method (°)
**226–240**	9.82 ± 3.79	11.74 ± 4.62
**244–257**	8.55 ± 2.77	12.12 ± 5.12

The tilt angle are obtained from the 1μs AA MD run at P = 1.01325 bar and T = 310 K and the 5 μs CG MD runs at P = 1.01325 bar and T = 310 K.

The tilt angles for AA and CG methods are remarkably similar, as shown in Table 4. The difference observed in the tilt angle of the entire helix is explained by the H5 torsion at the P244, which is more important for the CG system than for the AA one.

Although the CG is more rigid than the AA system, it can be seen that the CG model is sufficiently close to the results obtained for an AA system; it confirms the interest of using the CG simulations compared to the AA simulations.

### Tilt angle of the μOR helices

As already mentioned in the previous part, an important aspect of the GPCR conformations is their helix tilt angles (Table 5). A detailed analysis of the tilt angle of μOR in each membrane, and particularly, the tilt of each helix, shows that both entities of the supramolecular complex adapt one to each other by minimizing their polar contacts.

**Table 5:** Maximum tilt angles, with standard deviations, of each helix of μOR.

Helix	POPC	DPPC	POPE
**H1**	17.1 ± 1.7	18.7 ± 0.7	18.1 ± 0.8
**H2**	14.2 ± 1.0	15.0 ± 0.9	14.6 ± 0.9
**H3**	15.2 ± 0.8	15.3 ± 0.9	14.8 ± 0.5
**H4**	19.5 ± 0.8	18.4 ± 2.2	16.6 ± 0.4
**H5**	55.7 ± 7.1	63.7 ± 5.0	49.8 ± 9.5
**H6**	17.1 ± 1.6	19.6 ± 0.8	19.3 ± 0.8
**H7**	15.2 ± 0.7	14.2 ± 1.2	14.7 ± 1.3
**H8**	9.8 ± 0.3	10.1 ± 0.1	9.8 ± 0.5

They are calculated using the Bendix plugin of VMD for POPC, DPPC, and POPE systems, as obtained from the 15 μs CG MD runs at P = 1.01325 bar and T = 310 K.

All three types of lipids have a similar effect on the μOR conformations. Indeed, the tilt angle values varies between 10 and 20°, except for helix H5 which is characterized by a larger range of angle values, *i*.*e*., from 40 to 70°. Error bars for helix H5 are comprised between 5 and 9.5°, whereas for the other helices, they are between 0.1 and 2.2°. Helix H5 is one of the longest helix with 32 AAs, as well as in H1, H3, and H6. H5 has a marked flexibility due to the kink proline Pro 295 and longer extra- and intracellular loops compared to the other helices (Fig 1). The larger tilt angle value and the high degree of freedom of H5 allow to minimize the polar AAs exposition towards the lipid environment. Table 5 also shows that the H5 tilt is smaller for μOR embedded in POPE and larger in DPPC compared to our reference lipid, POPC. Helix H5 is twisted at P244, the torsion zone is defined by A240 to P244. Table 6 indicates the results obtained for the tilt angles of the first and second part of the H5, defined by W226—A240 and P244—L257.

**Table 6:** Tilt angle of the two part of helix H5 with standard deviation.

AA begin—AA end (H5)	POPC (°)	DPPC (°)	POPE (°)
**W226—A240**	11.79 ± 0.16	12.60 ± 0.71	11.84 ± 0.94
**P244—L257**	11.47± 0.62	15.35 ± 6.91	12.2 ± 0.45

They are calculated using the Bendix plugin of VMD for POPC, DPPC, and POPE systems, as obtained from the 15 μs CG MD runs at P = 1.01325 bar and T = 310 K.

Table 6 shows that the inclination of the helix H5 is larger for the second half of the helix (P244—L257), which is illustrated by the average tilt angle value of 15.35° instead of 11.47° and 12.2° for the POPC and the POPE respectively. The DPPC tail is shorter than the POPC ones (Fig 2). The thickness of the DPPC membrane is smaller (38 Å for DPPC and 42 Å for POPC). Helix H5 is thus more inclined and the protein is compressed in the membrane. These first results demonstrate the role of each lipid towards the protein conformations.

### Effect of the lipid structure on the number of interactions with the μ opioid receptor

**Fig 3:**
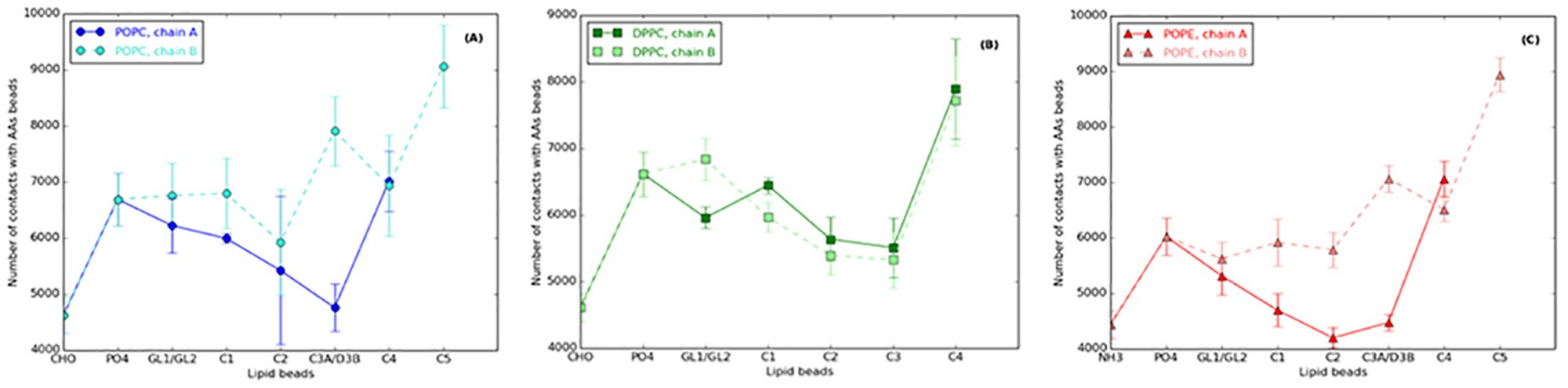
Number of contacts between each protein bead and each lipid bead type. **(A)** POPC, **(B)** DPPC, and **(C)** POPE, as obtained from the 15 μs CG MD runs at P = 1.01325 bar and T = 310 K. The D3B bead in POPC and POPE is replaced by a C3B in DPPC. Lines between the points are present to guide the reading of the graphs.

Each lipid interacts differently with μOR depending on their structure. To understand the effects of the different parts, for example the lipid heads (regarding choline or ethanolamine) and the acyl chain (regarding the saturated or unsaturated chain), we counted the contacts between each μOR bead and each lipid bead along each MD run (Fig 3).

From Fig 3, it is first seen, that for all lipids, the CHO/NH3 beads form less contacts with μOR than the PO4 one. It is due to the negative charge brought by PO4, which interacts with the positively-charged μOR surface AAs, contrarily to the CHO/NH3 beads that are positively charged.

The differences in the contact numbers involving the lipid acyl chain beads are not clearly marked. Indeed, error bars are relatively large and prevent chain A to be strictly differentiated from chain B, particularly from the GL to C2 beads for POPC and DPPC (Fig 2). There are less contacts with μOR for POPE than POPC from PO4 (with 6,026 and 6,691 contacts, respectively) to C2B (with 5,788 and 5,931, respectively) and C2A (4,198 and 5,423 contacts, respectively). The two chains of POPE behave differently regarding the number of contacts they form with the receptor. A strong and selective interaction between μOR and POPC for the D3B beads is observed, about 7,910 contacts compared to μOR and POPE, about 7,068 contacts. In all other cases, C3 beads form fewer contacts with μOR, *i*.*e*., only 5,328 contacts are formed between the protein and C3B in DPPC (Fig 3B). It is actually due to the unsaturated bead D3B in POPC (Fig 3), which involves a *cis* double bond with an average C2B-C3B-C4B angle of 120°, and a decrease of the lipid chain stiffness. The membrane fluidity that is affected by the lipid chain stiffness induces protein structural changes and a larger variety of μOR conformations. Hence, POPC interacts more easily with the μOR cavities that are formed during the simulations. It is thus assumed that particular lipid sites exist at the surface of μOR, which promote interactions with POPC due to its tail B flexibility.

All end beads, *i*.*e*., C4A/C4B/C5B in POPC and POPE and C4A/C4B in DPPC, are characterized by large numbers of contacts with the μOR AAs, above 7,000, *versus* most of the other lipid beads. Those end beads are located in the middle of the membrane and they interact with apolar AAs in the mid-part of the helices. The helices shift relative to the AAs in the middle, so that they move less and are more available for protein-lipid interactions.

The number of contacts involving the NH3 beads is 5% smaller than for the CHO beads but again it is not significant considering the error bars (Fig 3C). Contrarily, the difference regarding PO4 beads is significant as the total number of μOR-POPC contacts and μOR-DPPC contacts are 11% larger than for μOR-POPE; hence differences in interactions appear between PO4 and C2A/C2B. The ethanolamine beads lead to larger conformational changes, such that the first beads of POPE (PO4 to C2 beads) cannot interact as frequently as in the POPC membrane, the reference lipid. The contact number increases at the level of the last tail beads. The head beads, located at the interface protein/lipid/water, do not modify the interactions at the interface but ethanolamine decreases the number of contacts with the lipid beads comprised between PO4 and C2 compared to choline in POPC. C4 and C5 beads present exactly the same profile of interaction in POPE and in the lipid reference POPC.

In the next part of the paper, we will investigate more specifically each interaction between the lipids and every AA in order to identify more precisely the lipid interaction sites and their dependence on the head type and the acyl chain.

### Interaction profiles between POPC, POPE, and DPPC models and μOR

**Fig 4:**
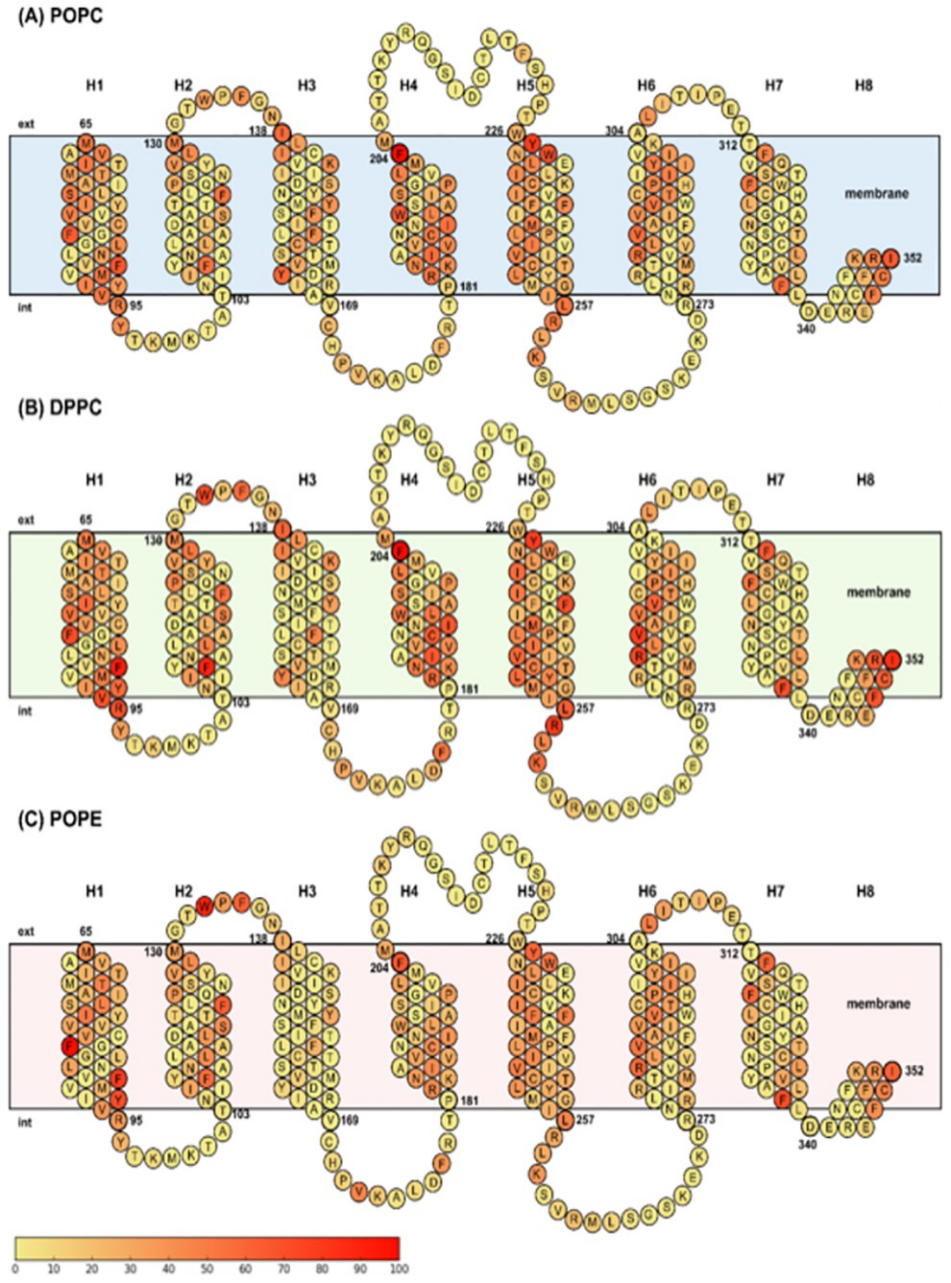
Primary structure of μOR in (A) POPC, (B) DPPC, and (C) POPE membrane. Each AA of μOR is colored as a function of its contact frequency with the lipids, as obtained from the 15 μs CG MD runs at P = 1.01325 bar and T = 310 K, ranging from yellow (0%) to red (100%).

During the MD simulation, the dynamic nature of the lipid-protein interactions is such as lipids interact with the protein only for a limited period of time. To specifically focus on the μOR interaction sites that are accessible to the lipids, we determined the interaction time as a function of the number of contacts between all lipid molecules and the μOR beads. The obtained values were further expressed as percentages *versus* the largest number of contacts observed between each of the AAs and the surrounding lipids (Fig 4). For a clearer reading of the figure, each AA is color-coded according to its interaction frequency with the lipid beads.

Firstly, the analysis of the contact numbers of each helix shows that helices H1, H4, and H5 interact more often (more AAs colored in red) with the three types of lipids (Fig 4). Several AAs in these three helices show contacts with all lipid types, *i*.*e*., H1 (F84, F87), H4 (F204), and H5 (Y227). Some AAs located in the other helices also frequently interact with all three lipid types, *i*.*e*., H2 (F108, F123), H6 (R280, V284), H7 (F320, F338), and H8 (I352). These AAs are mainly located in the membrane with the exception of F204 (H4) and Y227 (H5) that are found at the interface, *i*.*e*., the part of the protein in contact with both water and lipid heads.

Among apolar AAs, phenylalanines interact half of the simulation time with the three types of lipids, except F241 (H5) and F289 (H6) that are located in the middle of the helices. F241, in helix H5, is located below F237 and F239, those two interacting frequently with the lipids. Steric hindrance decreases the possibility for the lipid to interact with F241. In H6, F289 is in the hydrophobic core of the protein, with 8% of μOR-lipid contacts (Fig 4).

Regarding POPC and POPE, which differ at the level of their choline and ethanolamine heads (Fig 4A and 4C), for POPC, we note that contacts between the head beads and μOR occur at the level of H1 (M65, I69, S76, M90, V94), H3 (I138, Y166), H4 (R182, I186), and H5 (L257), while for POPE, contacts are found with H1 (I77, Y91) and H7 (F313). Several AAs of H3 (I138, Y166) and H7 (F313) of μOR interact specifically with POPC and POPE, respectively, as confirmed in the next part of the paper regarding lipid site analysis.

Helices H1 and H4 present two distinct interaction profiles depending on the membrane composition (Fig 4A and 4C). For H1, the most frequent contacts occur with AAs located at different places along the helix as the membrane changes, while for H4, contacts occur with the same AAs but the contact frequencies are larger for POPC than POPE. As already shown before, the contacts with lipids are more frequent for the AAs located in the middle of each helix, *e*.*g*., for C190, W192, and I193 in the POPE case (Fig 4C).

Fig 4 also shows that some AAs located at the protein/lipid/water interface act as potent contact sites with the lipid heads. For example, F204 in H4 and Y227 in H5 are characterized by frequent contacts with POPC and POPE. The contact frequency is smaller with POPE (66 and 61%, respectively) than with POPC (100 and 79%, respectively). Some AAs interact preferentially with choline in POPC, *i*.*e*., M65, I69, S76, M90, V94, I138, I176, R180, and L257 (Fig 4A) or ethanolamine in POPE, *i*.*e*., Y91, W133, F135, and F313 (Fig 4C). Thus, choline beads involve more contacts with the smaller AAs (methionine, isoleucine, serine, arginine, valine, and leucine) whereas ethanolamine frequently interacts with aromatic AAs (phenylalanine, tyrosine, and tryptophane) whose steric hindrance is larger. Indeed, choline is a larger bead than ethanolamine in the MARTINI description. As discussed before, the head type does not significantly affect the contact frequency of the CHO and NH3 beads while the head size involves less numerous contacts with beads PO4 to C2 for POPE than for POPC (Fig 3). We will further show that the lipid beads PO4 to C2 in POPE can actually not interact with the nearby AAs due to the ethanolamine bead contacts.

In order to understand the effects of the lipid acyl chain on μOR, we analyzed the contact frequencies occurring with POPC (Fig 4A) and DPPC (Fig 4B), *i*.*e*., the two lipids differing at the level of their chain B, with a C3B bead in DPPC or a D3B bead (representing unsaturation) in POPC (Fig 2).

We first restricted our study to contacts made with the AAs in the middle of the protein. Fig 4 shows that the AAs characterized by the highest contact frequencies occur at different positions at the level of H1 (S76) and H6 (V284) in POPC, and in H1 (I77, V80), H4 (L194), H5 (I234, F237, I238, M243, I247) and H6 (V284) in DPPC. There is thus a different distribution of contacts between μOR and either POPC or DPPC, S76 replaces I77 and V80 in H1, and V284 replaces V291 in H6. DPPC forms more contacts with helices H4 and H5 than POPC (10% more for H4 and 10 to 30% more for H5), even if most of the involved AAs are identical. Table 5 showed that the D3B bead in POPC presents more contacts with μOR than C3B in DPPC. With the unsaturated lipid, the membrane is more fluid and the lipid movements are larger. The fluidity of the membrane thus allows to increase the number of contacts with the μOR AAs, while a more rigid membrane constraints the protein, which promotes μOR-lipid contact interfaces.

POPC has one extra bead, C5B, at the end of the acyl chain B (Fig 2). Hence, a POPC membrane is thicker (42 Å) than a DPPC membrane (38 Å). To minimize contacts between apolar AAs and the polar environment, μOR adopts a more compact conformation, which can be characterized in terms of the helix tilt angles. As seen before, the H5 tilt angle is the most affected when the membrane type changes (Table 5). It is corroborated by the analysis of the contacts between the interface AA beads and the lipid beads. As shown in Fig 4A and 4B, the contacts between the lipids and the interface AAs are, for POPC, H1 (I69), H3 (Y166), and H5 (W228), and for DPPC, H1 (Y91, R95), H2 (L129), H5 (V250, L254, R258, K260), and H8 (R348, F350, C351). It is also seen that DPPC interacts more than POPC with the AAs of loop EL1, *i*.*e*., W133 and F135. There is an increase of 10% in the contact frequency of μOR with POPC *versus* DPPC.

The AAs at the protein/lipid/water interface interact thus differently with the three lipids, meaning there are preferred interfaces to inforce interactions between μOR and each lipid. In the following part, we will explore the path of each lipid around the protein to demonstrate how lipids move and block μOR in specific conformations.

### Lipid exchange in their sites at the protein surface

**Fig 5:**
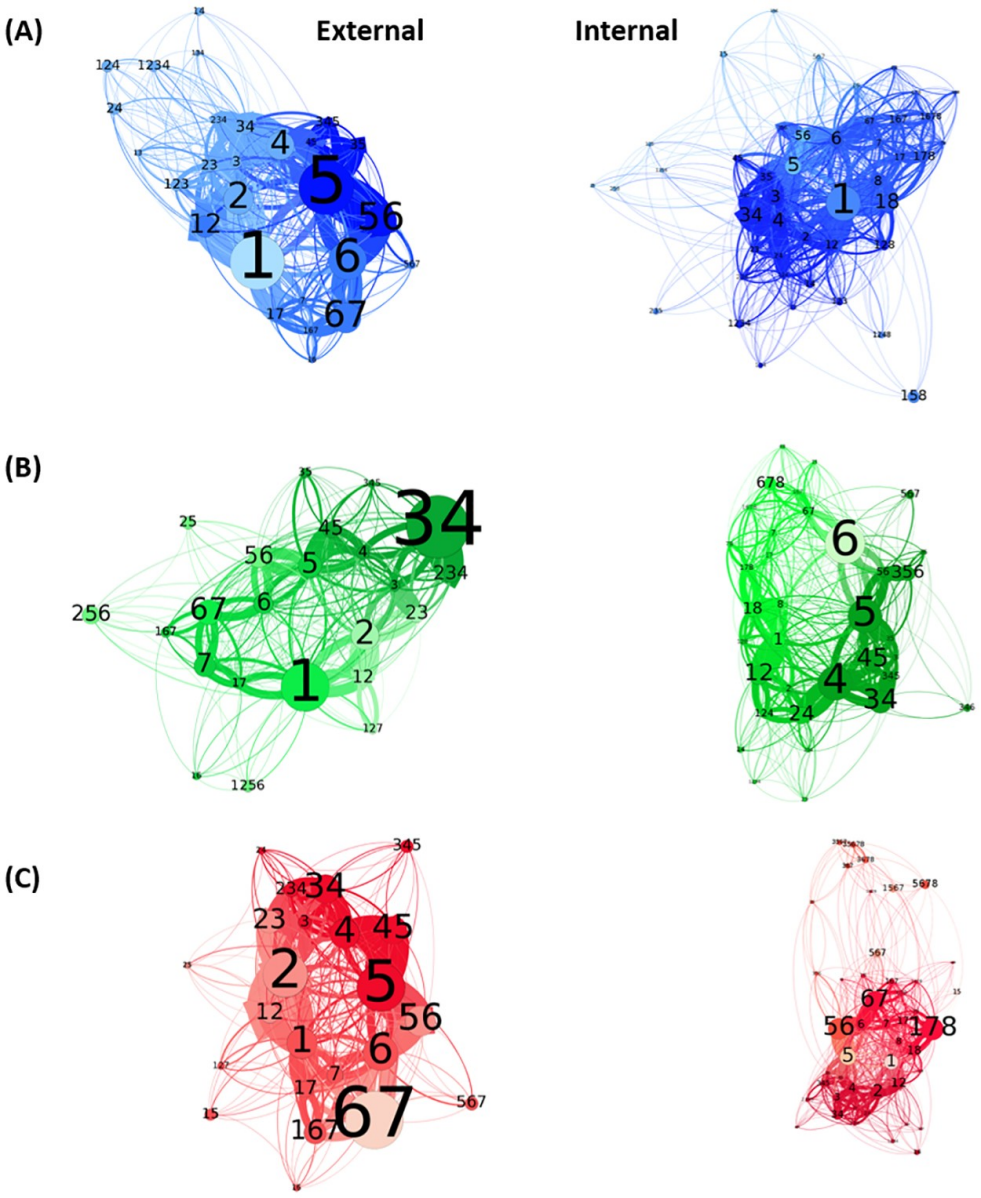
Network graph of lipid movements around μOR. Network graph obtained using Gephi as determined from the three 15 μs CG MD runs at P = 1.01325 bar and T = 310 K of (A) POPC, (B) DPPC, and (C) POPE interacting with μOR for the external and internal membrane layers. Each circle represents a site of μOR, according to helix label. As an example, 167 stands for helices H1, H6, and H7. The more often a site is visited by lipids, the larger the circle. In the same way, a connection between two nodes is thicker when the lipid transfer frequency between two protein sites is higher. In each network, the node color shade is a function of the clustering results, as described in Materials and Methods.

After having identified the AAs interacting with each type of lipid membrane, we studied which protein sites are preferentially visited by the lipid molecules. Indeed, despite the protein rigidity inferred by the MARTINI model, lipids are free to move and exchange their position in the 3D space. In the present section, we focus on the lipid shell surrounding the membrane protein at less than 0.5 nm of distance, both in the internal and in the external layers of the membrane. The preferred contact sites of the lipids and the paths they follow at the protein surface are visualized in Fig 5.

Fig 5 shows that helices H1 and H6 are privileged sites for all *annulus* lipids. As already mentioned in Part 3.3, there is a larger contact frequency between the lipids and the AAs of helix H1 (Fig 4). As also shown by the Gephi networks, to move from one major site to another, the lipids interact with intermediate sites such as 12, 18, 167, 56, and 67. We detect four clusters for all lipids colored by Gephi clustering as described in Methods, as described below, except for the internal layer of POPE with five clusters. These clusters allow to emphasize major sites (depicted with large circles) and to gather intermediate sites.

A comparison of the POPC and POPE molecular movements around μOR shows the sites often occupied by lipids, *i*.*e*., for POPC membrane, sites 1 and 5 for the outer layer and site 1 for the internal layer (Fig 5A), for POPE, site 67 in the external layer and sites 178, 56, and 67 for the external layer (Fig 5C).

In the particular case of site 1 occurring in the outer layer of POPC (Fig 5A), ethanolamine interacts with H1 and with sites either composed of the helices H2, H6 or H7. The AAs interacting with each lipid are located differently in H1 depending on the lipid nature. More precisely, choline interacts with M65, I69, and S76 (Fig 4A), while ethanolamine interacts with T70 and L74 (Fig 4C). The interactions with ethanolamine beads in H1 are less favorable than choline because POPE interacts more often with site 67 since helices H6 and H7 are close to H1. Regarding H6, ethanolamine beads interacts more with L305 than choline but lateral chains of POPC form more contacts with the AAs (Fig 4A). Regarding H7, F313 interacts more often with POPE than POPC and F320 interacts as often with POPC than POPE (Fig 4). One thus concludes that choline preferentially occurs in site 1 while ethanolamine is favorably in contact with μOR at the level of site 67 (Fig 5A and 5C). For both lipids, the lateral chains are linked to site 67.

Regarding site 5 of the external membrane layer, more interactions are detected between μOR and the POPC head beads than with the POPE head beads. They occur at the level of Y227 and W228 (Fig 4A). A reverse trend is observed with the lateral chain beads of both lipid types. Site 5 is an important site for both lipids with privileged contacts for choline and lateral chains of POPE. On the whole, ethanolamine interacts with site 67, and lateral chains both with sites 5 and 67 (Fig 5A and 5C).

Regarding site 4 of μOR, which is characterized by more contacts with POPC than with POPE, interactions especially occur between F204 of H4 and the lipid head beads. The same contact pattern is seen with helix H3. However, differences appear at site 2, with more contacts for ethanolamine with W133 and F135 of EL1 (Fig 4C). Also, the lateral chains of both lipids form the same number of contacts with the AAs of H2. Choline interacts with H3 and H4 and ethanolamine with EL1. Lateral chains of POPE form contacts preferentially with H2, while POPC presents contacts with H2, H3, and H4 indifferently.

Turning now towards the interactions between μOR and the internal layer of the POPC and POPE membranes, we note that main interaction sites are sites 1 and 178, respectively (Fig 5A and 5C). Regarding H1, both lipids establish contacts with V94 and R95, while choline beads also form contacts with I93 and Y96. These differences induce different patterns for the lateral chains, *i*.*e*., M90 and N86 for POPC and Y91 and L88 for POPE (Fig 4A and 4C). Regarding H7, ethanolamine beads make more contacts with F338 than choline. Finally, AAs in H8 present the same interaction profile for POPC and POPE. Choline beads interact preferentially with H1 and ethanolamine with H7, these latter beads inducing a different pattern for the lateral chains in H1 and a conserved pattern for the ones in H8 (Fig 4A and 4C). This explains the main interaction site 1 for POPC and site 178 for POPE (Fig 5A and 5C).

Since ethanolamine interacts with Phe 338 in H7, lateral chains can also make contacts with H6. Site 67, formed by a combination of helices H6 and H7, is thus the second major site accessible to POPE at the μOR surface (Fig 5C). POPE heads and lateral beads interact with H7 and with helices H7/H8 or H6, respectively. As H5 is strongly tilted (Table 5), compared to the other helices, the lateral chains of the lipids can also interact with H5 and H6, at site 56. Contacts between choline and R258 and L257 in H5 are more frequent along the MD than with ethanolamine (Fig 4A and 4C).

The displacement of lipids from site 67 to site 4 is created by the formation of contacts which appear to be more favorable for POPC, *i*.*e*., the choline beads with N183, then the phosphate group with R182 and K184 in H4 and Y166 in H3 (Fig 4A and 4C). The interactions with the lateral chains for helices H2, H3, and H4 are identical for POPC and POPE.

Regarding the comparison between POPC and DPPC located in the external layer of the membrane (Fig 5A and 5B), we note that site 1 is a major interaction site for both lipids, with more interactions between POPC with helix H1 than for DPPC. A close number of contacts is observed with the AAs located at the interface, while it differs for the AAs inside the membrane. Indeed, less numerous contacts between S76 are detected with the unsaturated D3B bead of POPC (Fig 4A). The AAs located in the middle of the membrane, *i*.*e*., I77, V80, and L83, create contacts with the terminal beads C4A/B and C5A/B, as already shown in Fig 3A and 3B. Choline beads of POPC and DPPC interacts similarly with μOR.

From site 1, the lipids can move to site 2 (Fig 5A and 5B). It is achieved through an AA anchor, F123, which makes the same number of contacts with both lipids. Regarding the EL1 loop, we note more contacts with W133 and F135 with DPPC than with POPC, as in our comparison of POPC and POPE. Then, the first GL and C1 beads of the lateral chains interact weakly with AAs of H2, such as M130 and L129 for both POPC and DPPC. On the contrary, POPC form numerous contacts with H3 through I138, L139, K141, and I142, which is not observed for DPPC (Fig 4A and 4B). Site 34 is the main interaction site with the external layer of DPPC (Fig 5B). Yet, interactions with AAs of helix H4 are very weak for DPPC compared to POPC for all the AAs in the membrane, such as P201, L200, L194, I193, and W192. DPPC head bead contacts are partly preserved with F204 at the μOR interface. The contacts are more favorable for the DPPC choline beads with the AAs in EL1, than for POPC choline beads with H4. Lateral chains interact more favorably with H4 and H3 for POPC than for DPPC.

Regarding site 5 (Fig 5A and 5B), the contacts are more favorable with the AAs at the interface, *i*.*e*., Y227 and W228, interacting more often with POPC than with DPPC (Fig 4A and 4B), whereas in the middle of the membrane, three AAs, I234, F237, and I238, frequently interact with the lateral chains of DPPC. Regarding H6, we note a different interaction profile for POPC and DPPC. Indeed, for DPPC, contacts are formed with AAs at the interface, like L305, while for POPC, contacts occur with AAs in the membrane, such as Y299, I298, P295, T294, and V291 (Fig 4A and 4B). Finally, for helix H7, two AAs interact equally with both lipids, *i*.*e*., F313 and F320. In addition, choline beads of DPPC interact with H6 and POPC with H5, while the lateral chains of DPPC interacts with H5 and POPC with H6 (Fig 4A and 4B).

In the last part of the analysis, we compare the POPC and DPPC behaviors at the level of the internal layer of the membranes (Fig 5B and 5B). Site 1 is the main interacting site for POPC, whereas for DPPC, it has a minor importance. In the more frequently visited sites, it is combined with other helices such as sites 12 and 18. R95, V94, Y91, and F87 in helix H1, interact more often with DPPC than with POPC, while the opposite trend is observed for Y96 and I93. Regarding H2 and H8, the number of contacts is larger for DPPC than it is for POPC. For H2, it involves F108 while for H8, it involves R348, F350, C351, and I352 (Fig 4A and 4B). POPC make more contacts with AAs in helix H1 and less with H2 and H8, privileged by DPPC.

Helix H6 is the major interaction site between μOR and DPPC (Fig 5B). It involves AAs such as, R280, L283, and V284, which interact more often with DPPC than with POPC, as also observed for F338 in helix H7 (Fig 4A and 4B). The next important interacting sites of DPPC are sites 5 and 4, with again more interactions with DPPC than POPC. For helix H4, three AAs frequently interact with DPPC, *i*.*e*., I186, C190, and I193. DPPC can also interact with both helices as observed for the intermediate site 45 (Fig 5B). For DPPC, a minor contact site, namely site 35, is also seen in Fig 5B. It is characterized by more numerous contacts between POPC and μOR, especially at the level of Y166 in H3, than with DPPC. A more rigid membrane promote contacts between μOR and the lipids in the internal leaflet of the membrane.

## Conclusions and discussion

In our work, we performed three 5 μs coarse-grained (CG) molecular dynamics (MD) simulations to illustrate the influence of three types of lipid membranes (varying in head type, length, and unsaturation) on the μ opioid receptor (μOR) 3D structure. The analyses were based on the tilt angle of the μOR helices, on a frequency analysis of the contacts between the μOR amino acids (AAs) and lipid beads, and on a network analysis through Gephi. To our knowledge, the last part is a very original method to study lipid-protein interactions and follow the particular lipid paths around the protein with a network graph.

Our CG model was validated *versus* AA simulation, by comparing the main parameters such as the RMSD, the tilt angle of the helices and the properties of the membrane, of which we mention here only the area by lipid. Let us note that other methods, like ELNEDYN [40] and GoMartini [41], based on elastic networks have been proven as good tools to increase the elasticity of CG models.

On one hand, it is shown that the choline head (CHO) of POPC and ethanolamine head (NH3) in POPE do not significantly change the μOR-lipid interactions with the AAs at the protein/lipid/water interface. Rather, the number of contacts occurring between μOR and the beads of the lipid chains, from PO4 to C2 is lower, due to the steric hindrance of the lipid head. The choline CHO bead is larger than the ethanolamine NH3 bead and interacts preferentially with the smaller AAs, such as methionine, isoleucine, serine, arginine, valine or leucine. At the opposite, NH3 interacts preferentially with aromatic AAs like phenylalanine, tyrosine, and tryptophane (Fig 4). Interactions with the AAs of these aromatic AAs are either impossible with the acyl chain beads in POPE or differ considerably.

Additionally, as the helices pivot around their central AAs and the interactions with the central AAs are retained, the end beads, C4 and C5, interact identically for POPC and POPE. The modifications of the interaction types between μOR and the acyl beads, from PO4 to C2, induce the blocking of the protein in a limited number of specific conformations. For example, ethanolamine interacts differently with helix H1 or μOR than choline does. The number of contacts made with the AAs at the water/lipid/protein interface is decreased while the number of contacts between the terminal C4 and C5 lipid beads and the middle of the membrane is increased (Fig 3).

Moreover, the tilt angle of helix H5 is larger for the POPC membrane than for DPPC (Table 5) because the membrane is thicker whereas for the DPPC membrane, due to saturation, it is more compact. Within the inner layer of the membrane, DPPC makes more contacts with the μOR receptor than POPC. The inner part of the protein is less flexible and it helps a rigid lipid like DPPC to interact more frequently. The outer part, where the ligand pocket is located, is more mobile and more sensitive to environmental changes that modulate the receptor part of the protein.

Our results confirm the modulation of μOR conformations by lipids and show precisely the preferable locations of the AAs which interact with each of the lipid types. As we used three different lipids, the various interactions block μOR in different shapes illustrating the need of MD simulations of proteins in complex membranes with more than one lipid. Similar adaptations of GPCRs by the lipids have already been observed both experimentally [42] and theoretically [43, 44].

The main sites for oligomerization are H1/H2/H8 and H5/H6 as determined by MD [45] and X-ray crystallography [2] for μOR as well as a lot of GPCRs [46, 47]. Knowing that these helices are also involved in weak interactions with lipids and that lipids can promote GPCRs dimerization [48–50], the question of knowing how the lipid sites determined within our work are available for dimerization can be raised.

To conclude on the protein conformations mediated by the lipid types, we have shown that the lipid heads modify the conformations of the proteins through a small number of interactions. They act as a guide for the acyl chain at the protein surface with a preference for unsaturated chains as in POPC and POPE. Finally, the observed modifications open the way to new horizons in the study of the ligand binding pocket as lipids change the conformations of the entire protein and not only the protein surface. Let us add that a direct link between protein-lipid interactions and protein function has been recently published [51], which would be interesting to see confirmed by experimental studies. From all those recent works, we can affirm the need of various lipid membranes together for the study of the protein μ with MD methods to characterize the diversity of protein conformations.

## Supporting information

S1 Table Number of atoms (AA systems) or residues (CG systems) for μOR embedded in each membrane types. (PNG)

S2 Table Protocol of the all-atom MD simulations. NVT (constant Number, Volume, and Temperature) and NPT (constant Number, Pressure, and Temperature).
(PNG)

S1 Fig Primary structure of μOR in the POPC membrane with contacts between protein and lipid beads at a range of (A) 0.5 nm, (B) 0.6 nm, and (C) 0.7 nm. Each AA of μOR is colored as a function of its contact frequency with the lipids, as obtained from the 15 μs CG MD runs at P = 1.01325 bar and T = 310 K, ranging from yellow (0%) to red (100%).
(EPS)

## Abbreviations

3D: three-dimensional
AA: amino acid
CG: coarse-grained
DPPC: 1, 2-dipalmitoyl-sn-glycero-3-phosphocholine
EL: extracellular loop
FF: force field
GPCR: G protein-coupled receptor
Hi: helix i
IL: intracellular loop
MD: molecular dynamics
PDB: Protein Data Bank
POPC: 1-palmitoyl-2-oleoyl-sn-glycero-3-phosphocholine
POPE: 1-palmitoyl-2-oleoyl-sn-glycero-3-phosphoethanolamine
TM: transmembrane
μOR: μ opioid receptor
