# Supplementary figures and images for: Interaction of POPC, DPPC, and POPE with the μ opioid receptor: A coarse-grained molecular dynamics study
Source: PLoS One. 2019 Mar 14;14(3):e0213646. doi: 10.1371/journal.pone.0213646 (PMC6417715; doi:10.1371/journal.pone.0213646)

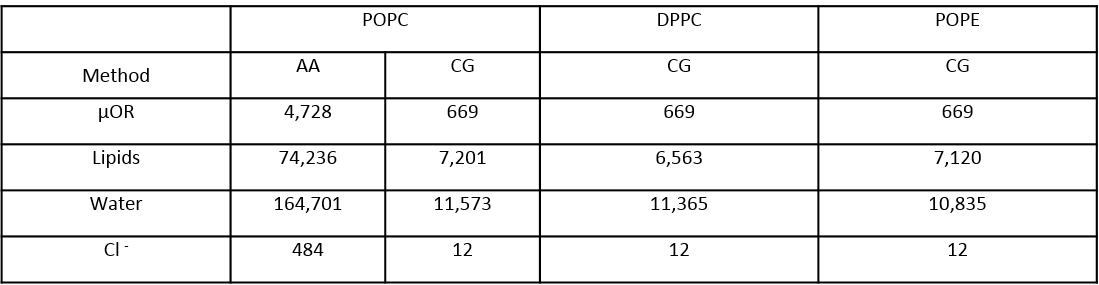

Supplement: S1 Table — (PNG) [file pone.0213646.s001.png]

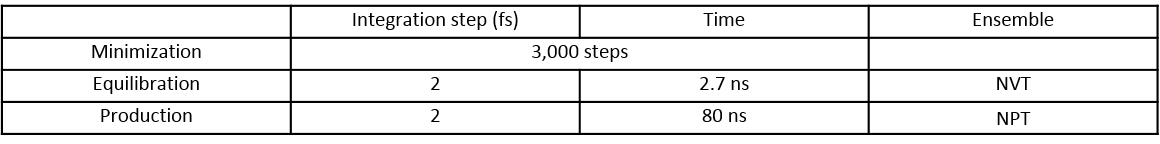

Supplement: S2 Table — NVT (constant Number, Volume, and Temperature) and NPT (constant Number, Pressure, and Temperature). (PNG) [file pone.0213646.s002.png]
